# The robust UCATR algorithm enhances the specificity and sensitivity to detect the infarct of acute ischaemic stroke within 6 hours of onset via non-contrast computed tomography images

**DOI:** 10.1186/s12883-022-02825-9

**Published:** 2022-08-04

**Authors:** Jianping Yu, Zhi Zhang, Qingping Xue, Tao He, Chun Luo, Kaimin Zhuo, Qian Yang, Tianzhu Xu, Jing Zhang, Fan Xu

**Affiliations:** 1 Department of Neurology, First Affiliated Hospital of Chengdu Medical College, Sichuan, 610500 China; 2 Department of Radiology, First Affiliated Hospital of Chengdu Medical College, Sichuan, 610500 China; 3 Department of Public Health, Chengdu Medical College, Sichuan, 610500 China; 4 MOEMIL Laboratory, School of Optoelectronic Information, University of Electronic Science and Technology of China, No. 4, Section 2, North Jianshe Road, Chengdu, 610054 China; 5 Department of Clinical Medicine, Chengdu Medical College, Sichuan, 610500 China

**Keywords:** Acute ischaemic stroke, UCATR algorithm, Non-contrast CT

## Abstract

**Problem background:**

Early detection of acute ischemic stroke (AIS) may provide patients with benefits against harmful health and financial impacts. The use of non-contrast computed tomography images for early detect of the infarct remains controversial.

**Materials & methods:**

Here, we used the UCATR algorithm to extract the pixel values of the infarct and the corresponding contralateral healthy area as the control surface in each NCCT slice for the whole brain. Magnetic resonance imaging results were used to verify both areas. We found significant pathological changes in the infarct compared with the corresponding contralateral healthy area in each NCCT slice.

**Attained results:**

Our approach validated that NCCT can be used to detect the lesion area in the early stage of AIS.

**Conclusions:**

With obvious advantages such as saving time and the ability to quantify the infarct volume, this approach could help more patients survive the fatal and irreversible pathological process of AIS and improve their quality of life after AIS treatment.

**Supplementary Information:**

The online version contains supplementary material available at 10.1186/s12883-022-02825-9.

## Introduction

Stroke is the second leading cause of mortality throughout the world, with 5.5 million deaths (95% uncertainty interval [UI] 5.3–5.7 deaths) [1]. It is also the second most common cause of disability-adjusted life years (DALYs). In China, stroke is the leading cause of death and acquired adult disability, presenting tremendous social and economic burdens. With a growing population and ageing, it is expected that the number of stroke patients and the health costs of stroke will continue to rise exponentially over time. Among the total prevalence of stroke patients, 84.4% (95% UI 82.1–86.4%) are ischaemic [2], usually caused by large artery atherosclerosis, cardioembolism, or small vessel occlusion. Consequently, implementing effective therapies against the onset of acute ischaemic stroke (AIS) is critical for further treatment and prognosis.

A previous study estimated that a total of 1.9 million neurons per minute are lost in an acute middle cerebral artery occlusion [3]. Given this speed, it is imperative to reduce the time required to diagnose AIS so that treatment can be started as soon as possible. This approach would optimise the post-AIS treatment outcome. Because rapid identification of the presence and extent of the infarct plays an essential role in AIS treatment, neuroimaging technology is an indispensable component to diagnose AIS and guide the subsequent therapies.

Magnetic resonance imaging (MRI), especially diffusion-weighted MRI (DW-MRI), is regarded as the most sensitive imaging modality to detect AIS, reaching 73–92% sensitivity in the first 3 h. Given that the benefits of AIS therapy are highly time sensitive, treatments should be initiated as soon as possible. Unfortunately, on average the MRI scanning process takes more than 15 min. Non-contrast computed tomography (NCCT) is the most efficient and time-saving neuroimaging modality prior to intravenous alteplase therapy, which is most effective when delivered to patients within 4.5 h of stroke onset. NCCT has a markedly lower sensitivity than MRI to detect AIS: 57–71% in the first 24 h and 12% in the first 3 h. However, the NCCT scan requires less than 10 s, a much shorter time than required by MRI. Therefore, improving NCCT sensitivity to detect AIS early after it occurs is essential to improve the health outcome of patients [4, 5].

Diagnosing AIS early by using NCCT is challenging because the density and texture variations in the brain are too subtle to show an infarct within 12–25 h after AIS. Indeed, the subtle changes in the NCCT images of stroke are too imperceptible for the human eyes to observe, particularly in the hyperacute stage. Fortunately, computer technology has provided an exciting advance, with the ability to capture subtle changes on images. Specifically, using machine learning to segment ASI infarcts based on NCCT images presents excellent concordance with the stroke volume on DW-MRI images [6].

With the rapid development of computer-based techniques to distinguish features of images, we can screen out single pixel changes in CT images. In this study, we aimed to increase the accuracy for detecting ischaemic lesions in NCCT images and to improve the prognosis of patients. We used computer-aided technology to assess infarcts in NCCT images that are invisible to the human eye within 6 h of stroke onset.

## Methods

### Subjects

We retrospectively reviewed patients with AIS who were admitted to the First Affiliated Hospital of Chengdu Medical College (Chengdu, China) from 1 January 2019 to 1 April 2019. The inclusion criteria were: (1) received a clinical diagnosis of AIS; (2) diagnosis was within 6 h of stroke onset; (3) anterior circulation ischaemic stroke due to large-vessel occlusion; and (4) no early signs of infarct lesions for the first NCCT scan after stroke onset. The exclusion criteria were: (1) no first NCCT scan available; (2) a significant early sign of infarct lesions for the first NCCT scan, which defined as slight decreased density in the infarction area, brain swelling, hyperdense middle cerebral artery sign, loss of insular ribbon, obscuration of lentiform nucleus, sulcal effacement.; (3) a history of stroke and intracranial malacia; (4) a history of central nervous system disease and left intracranial malacia; and (5) infarct could not be found based on CT or MRI 24 h after onset. Ten patients were included in our study. Diagnoses were confirmed independently by two experienced physicians via reviewing clinical, laboratory, and radiological files from electronic medical.

### Identifying potential infarcts by using CT and MRI images

**Fig. 1:**
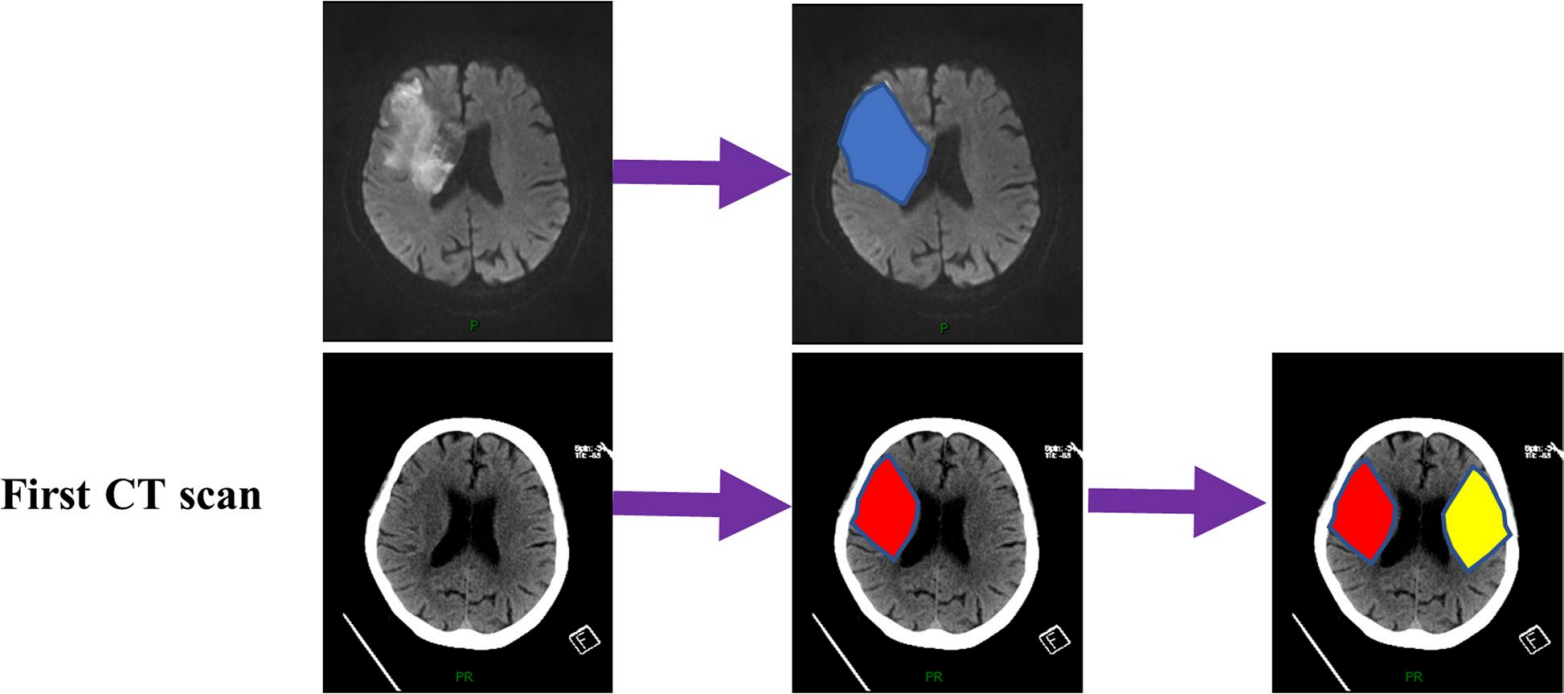
Workflow of pixel value extraction

Each patient was subjected to two neuroimaging examinations. First, each patient underwent the NCCT examination within 6 h and showed no significant early signs of an infarct. Second, each patient underwent an MRI examination, and there was evidence of an infarct. After collecting those neuroimages, two experienced physicians independently identified the infarcts on the MRI images, and they reached a consensus for a final decision. Then, two radiologists marked the infarct on the NCCT image as a red area. They marked the corresponding contralateral healthy area as a yellow area (Fig. 1).

### Methods for extracting pixel values

**Fig.2:**
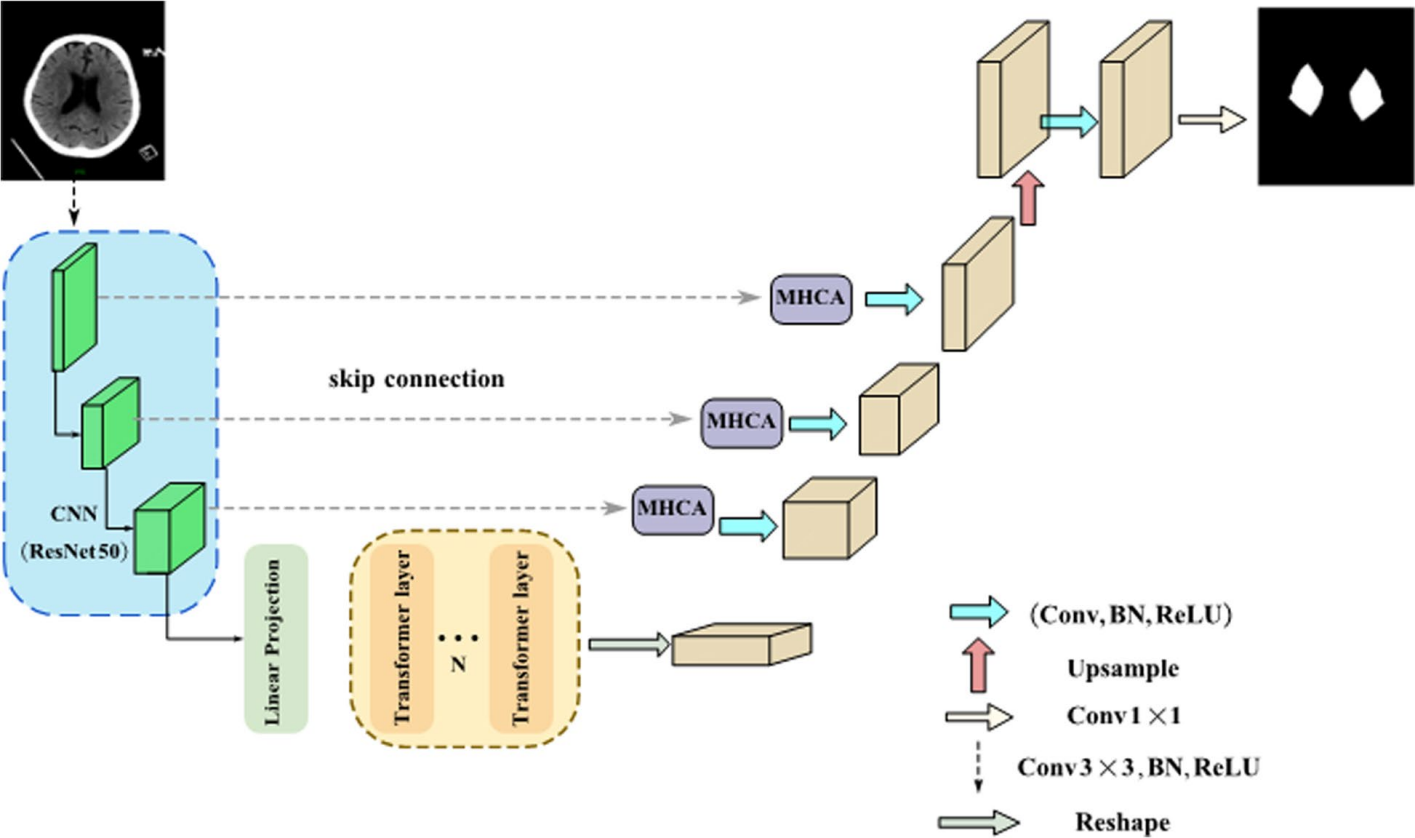
The architecture of UCATR to generate the mask

The pixel values of a potential infarct were extracted with the UCATR method, which uses the convolutional neural network (CNN) transformer encoder and cross attention decoder to effectively learn the global context features and suppress the unrelated or noisy areas. As shown in Fig. 2, we used ResNet50 as the CNN backbone to obtain the feature maps in different scales and then filtered out their irrelevant information by skip-connections and multi-head cross-attention (MHCA) modules to achieve high-precision spatial information recovery. For the NCCT images with a low signal-to-noise ratio, anisotropy of images, and anatomical asymmetry, MHCA modules were used in all positions of skip-connection. This approach ensured a better fusion of low-level features with high-level features, because the feature maps transmitted from the CNN through the top skip-connection have more original information and fewer semantic features, while the feature maps that result from middle and bottom skip-connection provide more abstract features. The transformer especially used at the bottom to learn global features and to provide long-distance structural information enables better recognition of small target lesions. Implementation of the end-to-end method allowed us to input a CT image and to generate a mask that directly indicates the significant difference in pixel values where a potential infarct lies and its corresponding contralateral healthy area. The pixel values were obtained by multiplying the original image by the mask.

### Statistics

Continuous variables are summarised with the mean and standard deviation (SD) while categorical variables are summarised with frequency and percentage. Because the pixel values failed to follow a normal distribution, the Wilcoxon test was used to compare the pixel values of an infarct in the first CT scan and its corresponding contralateral healthy area for each person and CT slice. Multilevel linear regressions were applied to estimate coefficients and 95% confidence intervals (CIs) for the difference in the pixel values between the infarct and the corresponding contralateral healthy area. For this analysis, the first level was the pixel value per person per CT image, the second level was the CT image per person, and the third level was the individual image. In the regressions, we adjusted for age, sex, and onset time. All analyses were conducted by using R version 3.5.1. *P* < 0.05 was defined as statistically significant.

## Results

### Patient characteristics

Table 1 shows the patient characteristics. For the 9 patients included in our research, the mean age was 77.80 years (SD = 6.46, range = 66–79 years), three were male, and the mean onset time was 4.49 h (SD = 3.28, range = 73–88 h). The TOAST classification was cardioembolism for nine patients and small-vessel occlusion for one patient.

**Table 1:** The basic characteristics of patients

No	Sex	Age	OCT(hour)	Culprit Artery	TOAST classification	NCCT slices
1	Male	79	1.77	LMCA	cardioembolism	17
2	Female	88	unknown	RMCA	cardioembolism	7
3	Male	66	6.27	RMCA	cardioembolism	24
4	Female	79	1.42	LMCA	cardioembolism	11
5	Female	81	2.03	LMCA	cardioembolism	42
6	Female	74	2.23	RICA	cardioembolism	30
7	Female	73	unknown	RMCA	cardioembolism	39
8	Female	86	3.88	LMCA	cardioembolism	16
9	Male	74	1.53	MICA	cardioembolism	37

*Abbreviation*: *OCT* time from onset to first CT scan, *NCCT* Nonenoncontrast computed tomography, *LMCA* Left middle cerebral artery, *RMCA* Right middle cerebral artery, *LICA* Left internal carotid artery, *RICA*, Right internal carotid artery. unknown, wake-up stroke

### The differences in pixel values between the infarct and the corresponding contralateral healthy area for each patient and CT slice

Supplementary Figs. 1, 2, 3, 4, 5, 6, 7, 8, 9 show the results for comparing the pixel values between the infarct and the corresponding contralateral healthy area for each patient (1–9) and CT scan. For seven patients (1, 3, 5, 6, 8, and 9), for most CT scans we found the pixel values of the infarct were significantly higher than the corresponding contralateral healthy area. However, patients 4 and 8 showed the reverse result. For patient 2, there was no significant difference in the pixel values of the infarct and the corresponding contralateral healthy area for any of the CT scans.

### The differences in pixel values between the infarct and the corresponding contralateral healthy area for each patient

Table 2 shows the differences in pixel values between the infarct and the contralateral healthy area for each patient. There were variable pixel values for both the infarct and the corresponding contralateral healthy area. Overall, the infarcts of nine patients had higher pixel values than the corresponding contralateral healthy area, and one patient showed the reverse result. Specifically, seven out of 9 patients showed that the pixel values in infarct were significantly higher than the corresponding contralateral healthy area (*P* < 0.001). However, patients 2 and 9 showed that the pixel values in the infarct were significantly lower than the corresponding contralateral healthy areas (*P* < 0.001).

**Table 2:** The differences of pixel values between infarction lesions and normal areas for each patient

Number	Normal	Infarction lesions	*P*^*a*^
	**Median (IQR)**	**Median (IQR)**	
1	85 (67, 100)	95 (76, 111)	< 0.001
2	71 (60, 85)	71 (61, 84)	0.82
3	96 (77, 113)	98 (79, 114)	< 0.001
4	83 (69, 97)	81 (67, 95)	< 0.001
5	96 (78, 113)	99 (80, 116)	< 0.001
6	85 (70, 99)	91 (77, 104)	< 0.001
7	71 (57, 99)	85 (57, 99)	< 0.001
8	93 (74, 110)	93 (75, 110)	< 0.001
9	87 (69, 105)	85 (70, 99)	< 0.001

*Abbreviation*: *IQR*, Inter-quartile range

^a^ *P* value was estimated by Wilcox tests

### The association between the infarcts and pixel values

Table 3 shows the results of multilevel regressions. We found that on average, the pixel values in infarcts increased by 3.70 compared with the corresponding contralateral healthy area. The pixel values on average were higher in female compared with male patients (*β* = 7.89, *P* < 0.001). The pixel values on average decreased 0.47 as the age increased by 1 year (*P* = 0.01). The pixel values on average decreased 0.91 as the stroke onset time increased by 1 h (*P* = 0.01). Compared with the patients with small-vessel occlusion, patients with cardioembolism had higher pixel values (*β* = 10.60, *P* < 0.001).

**Table 3:** The association between the infarction lesions and pixel values

Sample types	Beta coefficient	S.E	*P*
**Area**			
Normal	Ref		
Infarction lesions	3.71	0.04	< 0.001
**Sex**			
Male	Ref		
Female	11.83	0.90	< 0.001
**Age**	0.78	0.20	< 0.001
**Time**	-7.02	0.96	< 0.001
**Location**			
LMCA	Ref		
RMCA	49.76	7.15	< 0.001
Others	11.68	1.48	< 0.001

*Abbreviation*: *LMCA* Left middle cerebral artery, *RMCA* Right middle cerebral artery

## Discussion

We used the UCATR algorithm to identify potential infarcts based on NCCT images by using MRI images to verify and outline the infarcts and corresponding contralateral healthy areas. Working independently, two neurologists did not identify infarcts on the NCCT images during the hyperacute stage. When applying the UCATR algorithm, however, there were significant differences in the pixel values of infarcts compared with the corresponding contralateral healthy area based on the first CT scan. Seven out of 9 patients manifested significantly higher pixel values in the infarct compared with the corresponding contralateral healthy area. Furthermore, the three-level regression models confirmed these results after adjusting for age, sex, onset time, location, and type of AIS. Thus, the potential diagnostic capacity of AIS with NCCT images is compromised by the poor pixel discrimination abilities of human eyes.

Neuroimaging is the cornerstone for the diagnosis of suspected AIS. NCCT and MRI are mainstream diagnostic tools for AIS: they have been assessed extensively to rule out haemorrhage and conditions that mimic stroke and to select the best candidates for treatments. Though NCCT is the real-world imaging workhorse due to its broad availability, low cost, and cost-effectiveness [7], it suffers from lower sensitivity of identifying ischaemic lesions compared with MRI, especially DW-MRI, which is the reference standard for the detection of early ischaemia. Previous studies suggest that the signs of infarct on NCCT are not visible within the first 8 h after stroke onset [8]. Our research indicates that computer-aided diagnosis provides a promising improvement in assessing infarcts. While the two well-trained and qualified neurologists failed to observe the subtle changes in pixel values of NCCT images during the early stage of stroke, the computer-aided UCATR approach revealed that seven out of 9 stroke patients had higher pixel values in the infarct than the corresponding contralateral healthy area. Furthermore, we found that the pixel values in the infarct increased on average 3.70 compared with the corresponding contralateral healthy area after adjustment of potential confounders in multilevel regressions. A previous study used a computational method, the modified stroke imaging maker (SIM) method, to detect and localise invisible hyperacute ischaemia; the ischaemic hemisphere detection rate was 76%. This accumulated evidence suggests that the computer-aided method is sensitive in detecting subtle Hounsfield unit (HU) changes between brain hemispheres, even though the changes are imperceptible to trained neuroradiologists [9]. Both studies indicate that the ability of NCCT images to assess infarcts is underestimated because of the low ability of the human eye to distinguish changes. Therefore, the ability to detect the subtle changes in stroke NCCT images could be enhanced further by using advanced computational methods.

In a recent study, researchers collected NCCT images in patients with AIS (< 6 h from symptom onset to CT) who also underwent DW-MRI within 1 h. They found a good concordance between the machine learning approach for segmentation of infarcts due to AIS on NCCT images and stroke volume on DW-MRI scans [6]. In another study, the researchers localised the infarcts of hyperacute ischaemic stroke on NCCT images among 139 patients via image registration of their corresponding follow-up DW-MRI examination. Then, they implemented several supervised methods to capture the textural differences between the infarcts with their corresponding contralateral healthy areas [10]. Although we did not try to segment the infarcts in NCCT images for hyperacute ischaemic stroke, we rationalised those segmentations by using advanced computational methods. Because MRI allow for highly sensitive detection of infarcts, numerous methods have been developed to segment semi-automatically or fully automatically the infarct of stroke by using MRI. Our research, however, indicates the potential high values and benefits to develop and implement those methods based on NCCT images for assisting neuroradiologists to identify the early invisible infarct in AIS. Thus, additional methods should be developed to segment the invisible signs of early stroke in NCCT images to ensure optimum treatments and an improved prognosis. The mechanisms that underlie our results might relate to the early ischaemic changes in NCCT images, characterised by the presence of hypodense infarcts, but the NCCT images suffered from a low signal-to-noise ratio, anisotropy of images, and anatomical asymmetry [11]. Hence, the subtle changes in NCCT intensity values are imperceptible to neurologists during the first few hours from ictus [12].

To our knowledge, our study is the first attempt to compare the pixel values of infarcts that are invisible on the first CT scan and the corresponding contralateral healthy area. Our findings could strengthen the reliability of segmentation and detection of invisible infarcts based on NCCT images. The future work will be recruit more qualified samples to validated sensitivity and specificity of pixel value changes in NCCT scan in diagnosis against stroke.

## Conclusion

This pilot study has validated that the pixel values of an infarct induced by AIS can be detected effectively with NCCT images. Our findings have been validated by using MRI images to identify the infarcts and corresponding contralateral healthy areas.

### Limitations

First, because this study represents a pilot validation, we employed a small sample size. Second, we did not constrain the time between the stroke onset and MRI scans of the brain, a factor that might influence the size of the infarct identified on NCCT images. Because this might have an impact on our results, we adjusted the onset time in the multilevel regressions and found a significant difference between infarcts and corresponding contralateral healthy areas, a phenomenon that could to some extent validate our results. Third, considering all admitted patients who underwent intravenous thrombolysis or intravascular thrombectomy, the infarcts of second CT scans or MRI were smaller than the real ones, a factor that might have had an impact on our results.

## Supplementary Information

**Additional file 1: Supplementary Figure 1. **Comparing the pixel values between the infarct and the corresponding contralateral healthy area for patient 1# under the CT scan. **Supplementary Figure 2.** Comparing the pixel values between the infarct and the corresponding contralateral healthy area for patient 2# under the CT scan. **Supplementary Figure 3.** Comparing the pixel values between the infarct and the corresponding contralateral healthy area for patient 3# under the CT scan. **Supplementary Figure 4. **Comparing the pixel values between the infarct and the corresponding contralateral healthy area for patient 4# under the CT scan. **Supplementary Figure 5.** Comparing the pixel values between the infarct and the corresponding contralateral healthy area for patient 5# under the CT scan. **Supplementary Figure 6.** Comparing the pixel values between the infarct and the corresponding contralateral healthy area for patient 6# under the CT scan. **Supplementary Figure 7.** Comparing the pixel values between the infarct and the corresponding contralateral healthy area for patient 7# under the CT scan. **Supplementary Figure 8.** Comparing the pixel values between the infarct and the corresponding contralateral healthy area for patient 8# under the CT scan. **Supplementary Figure 9.** Comparing the pixel values between the infarct and the corresponding contralateral healthy area for patient 9# under the CT scan.

## Data Availability

All data would be made available upon reasonable request to corresponding author.
