# Supplementary material for: The robust UCATR algorithm enhances the specificity and sensitivity to detect the infarct of acute ischaemic stroke within 6 hours of onset via non-contrast computed tomography images
Source: BMC Neurol. 2022 Aug 4;22:291. doi: 10.1186/s12883-022-02825-9 (PMC9351169; doi:10.1186/s12883-022-02825-9)
Supplement: Supplementary file 1 — Additional file 1: Supplementary Figure 1. Comparing the pixel values between the infarct and the corresponding contralateral healthy area for patient 1# under the CT scan. Supplementary Figure 2. Comparing the pixel values between the infarct and the corresponding contralateral healthy area for patient 2# under the CT scan. Supplementary Figure 3. Comparing the pixel values between the infarct and the corresponding contralateral healthy area for patient 3# under the CT scan. Supplementary Figure 4. Comparing the pixel values between the infarct and the corresponding contralateral healthy area for patient 4# under the CT scan. Supplementary Figure 5. Comparing the pixel values between the infarct and the corresponding contralateral healthy area for patient 5# under the CT scan. Supplementary Figure 6. Comparing the pixel values between the infarct and the corresponding contralateral healthy area for patient 6# under the CT scan. Supplementary Figure 7. Comparing the pixel values between the infarct and the corresponding contralateral healthy area for patient 7# under the CT scan. Supplementary Figure 8. Comparing the pixel values between the infarct and the corresponding contralateral healthy area for patient 8# under the CT scan. Supplementary Figure 9. Comparing the pixel values between the infarct and the corresponding contralateral healthy area for patient 9# under the CT scan. [file 12883_2022_2825_MOESM1_ESM.docx]

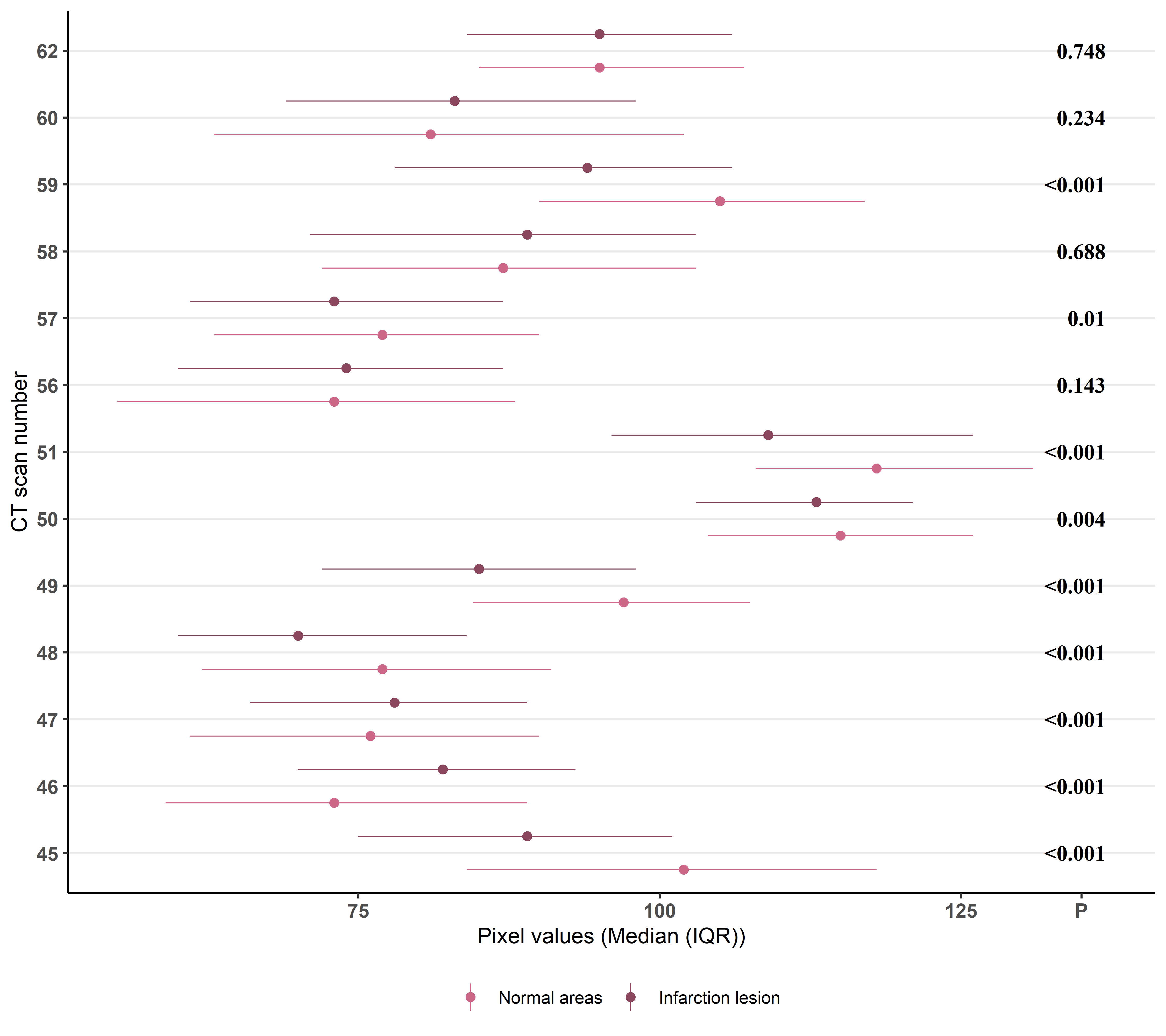


**Supplementary Figure 1:** Comparing the pixel values between the infarct and the corresponding contralateral healthy area for patient 1# under the CT scan.


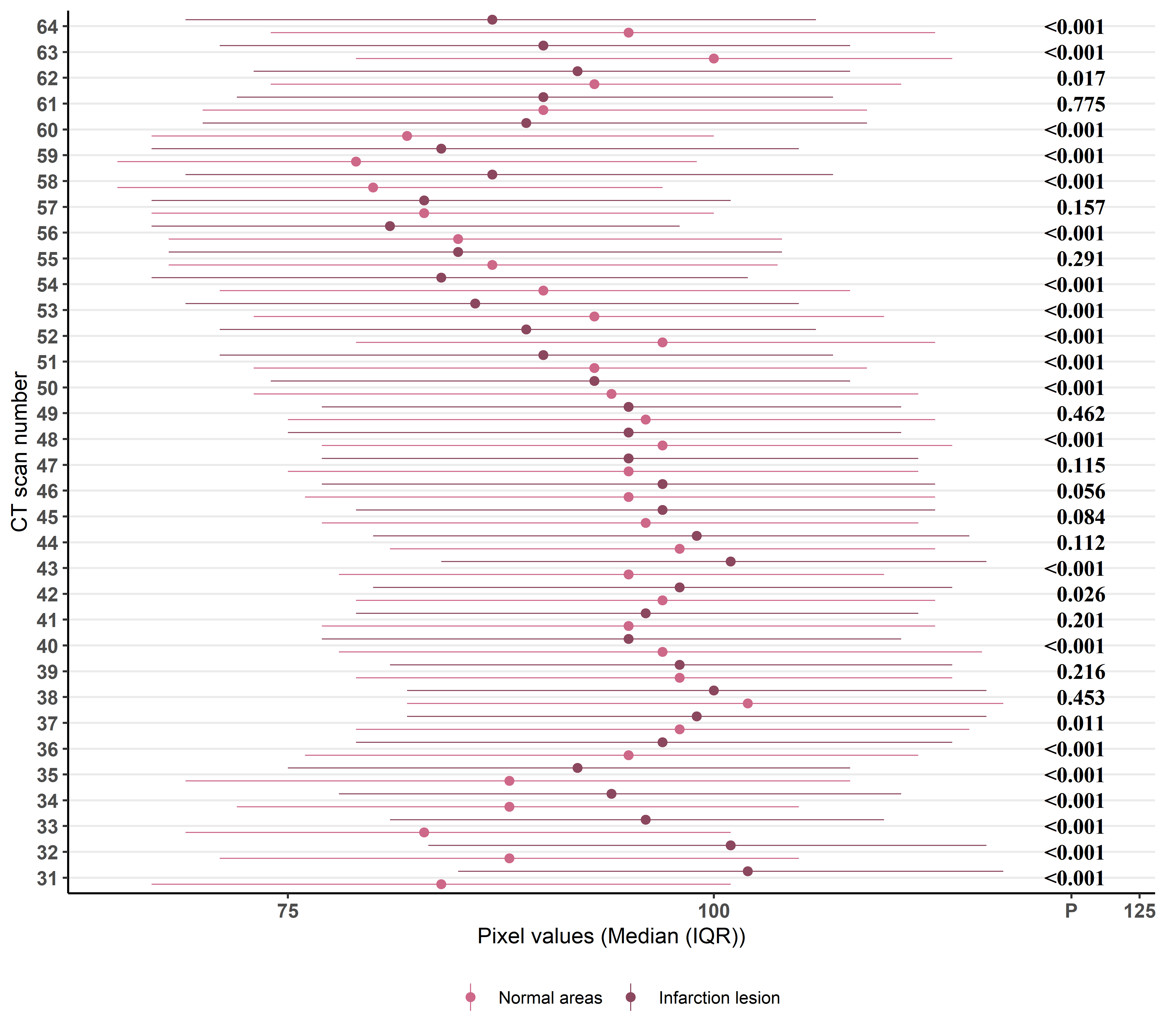


**Supplementary Figure 2:** Comparing the pixel values between the infarct and the corresponding contralateral healthy area for patient 2# under the CT scan.


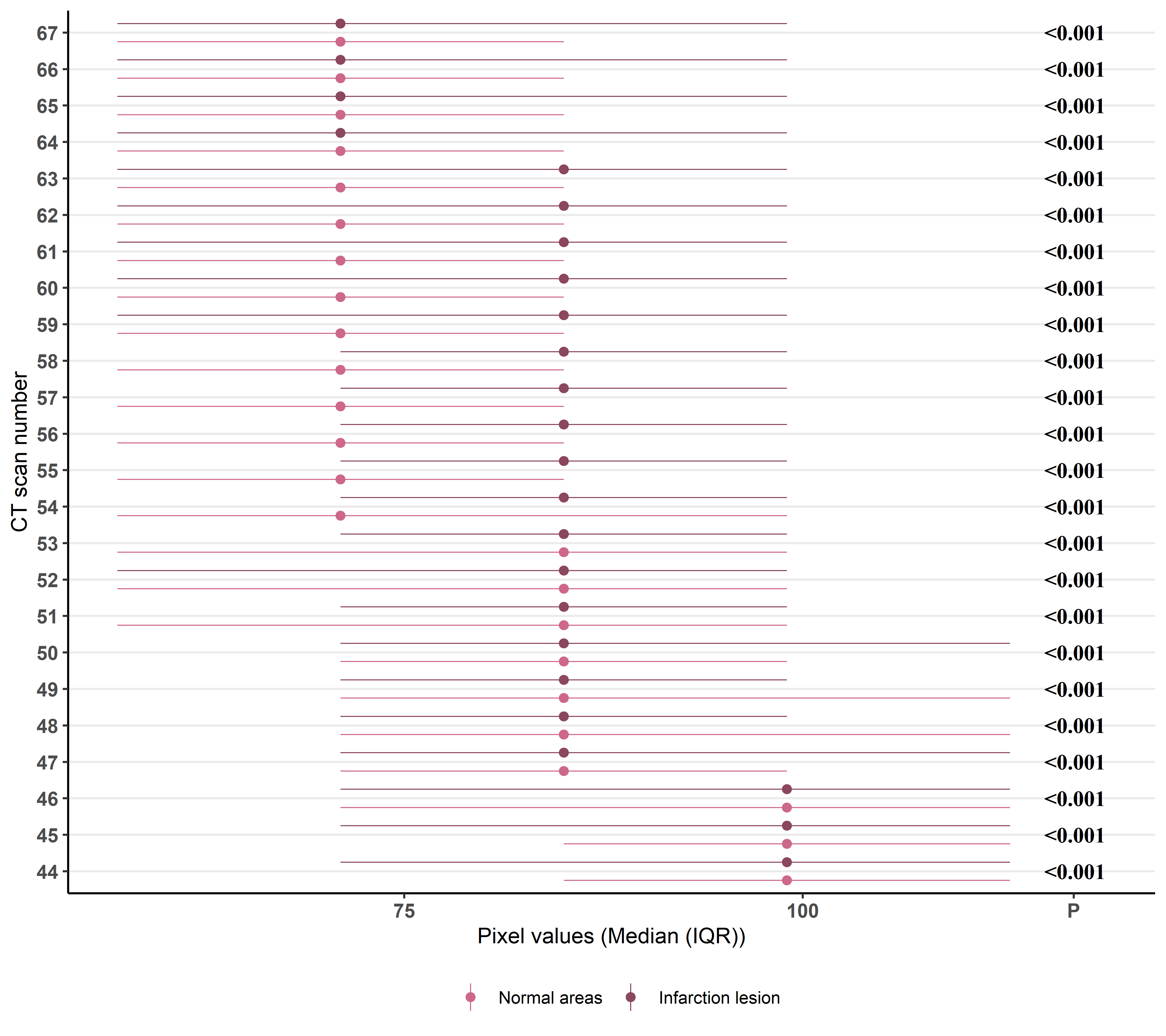
 **Supplementary Figure 3:** Comparing the pixel values between the infarct and the corresponding contralateral healthy area for patient 3# under the CT scan.


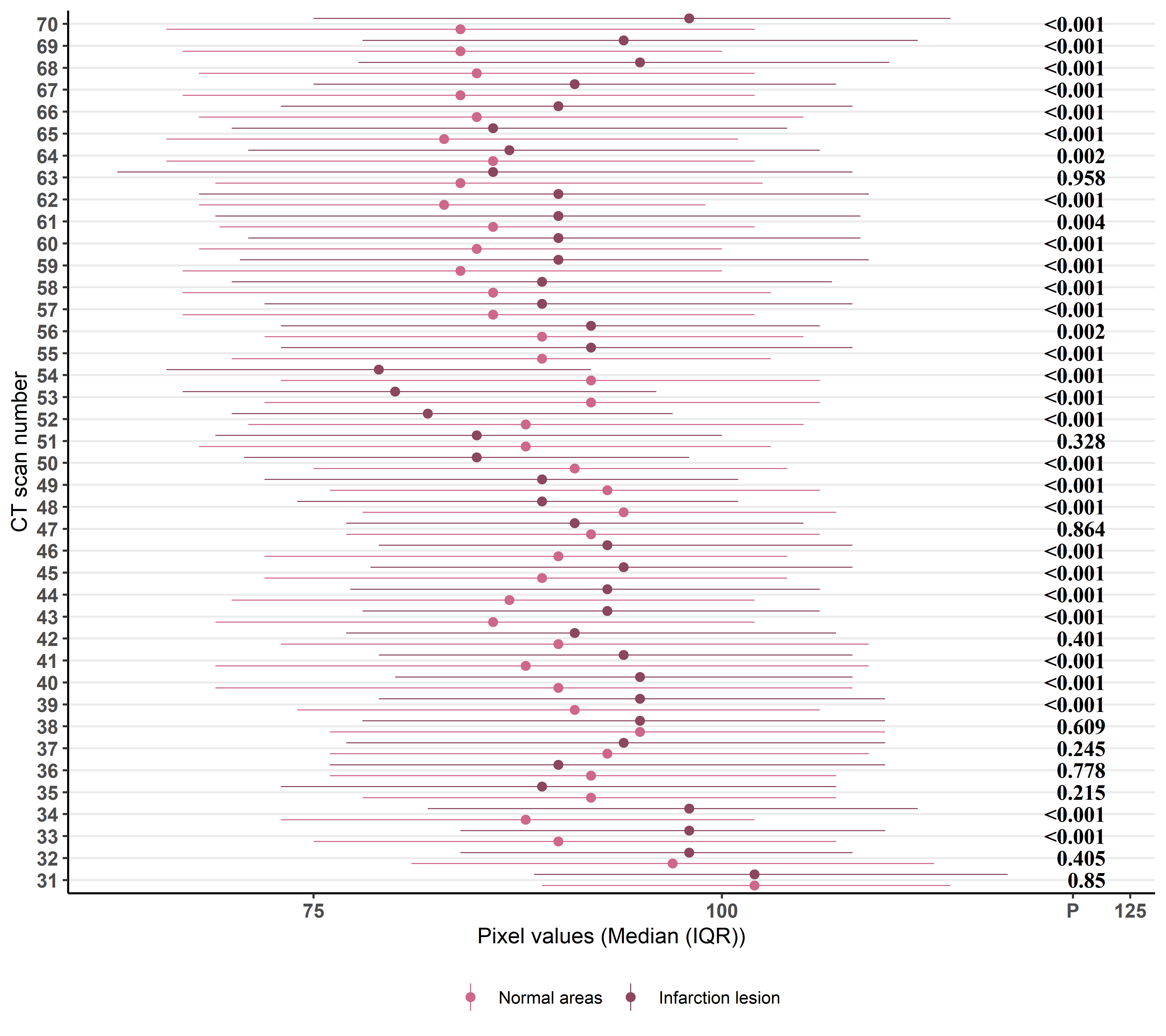


**Supplementary Figure 4:** Comparing the pixel values between the infarct and the corresponding contralateral healthy area for patient 4# under the CT scan.


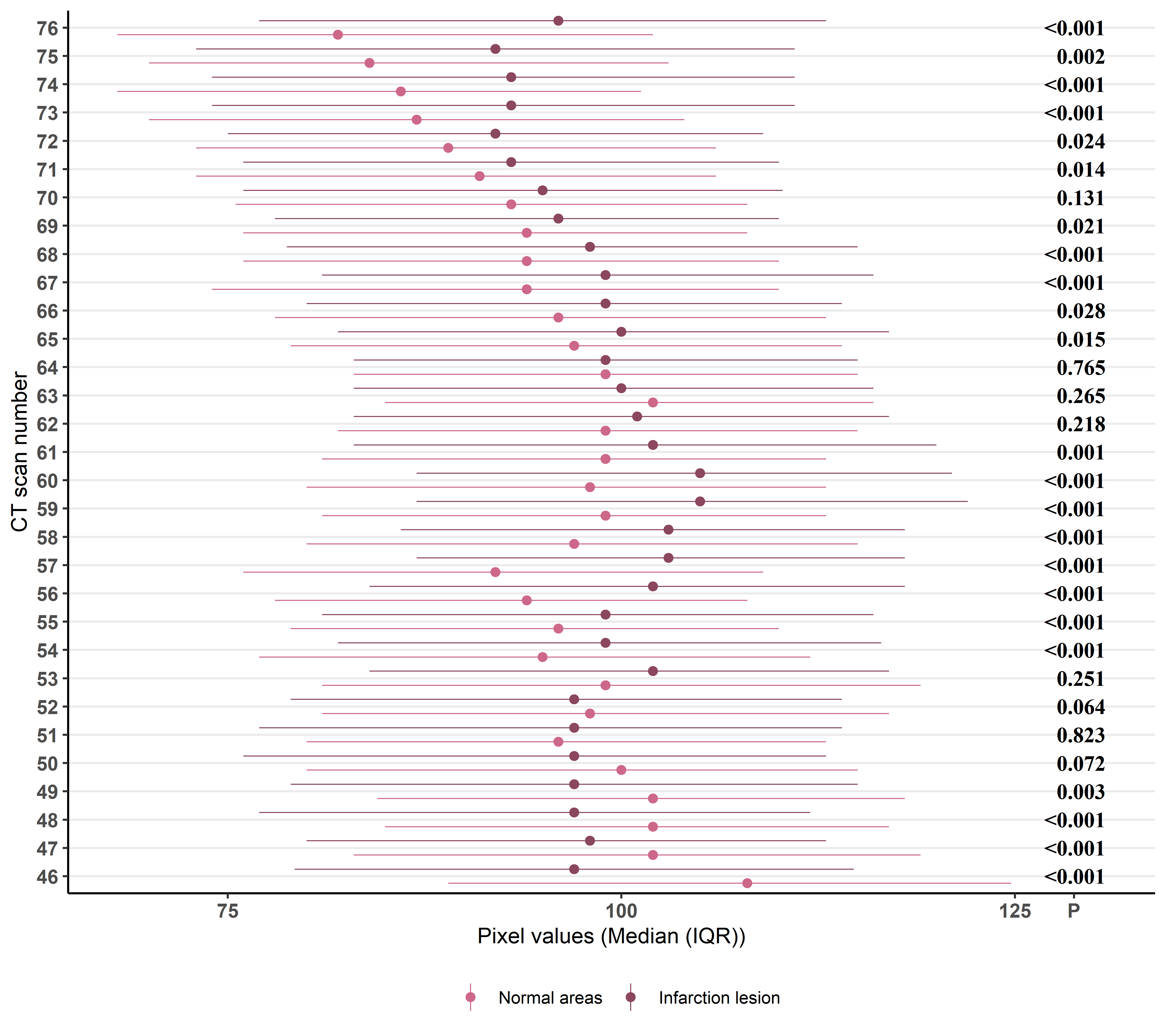


**Supplementary Figure 5:** Comparing the pixel values between the infarct and the corresponding contralateral healthy area for patient 5# under the CT scan.


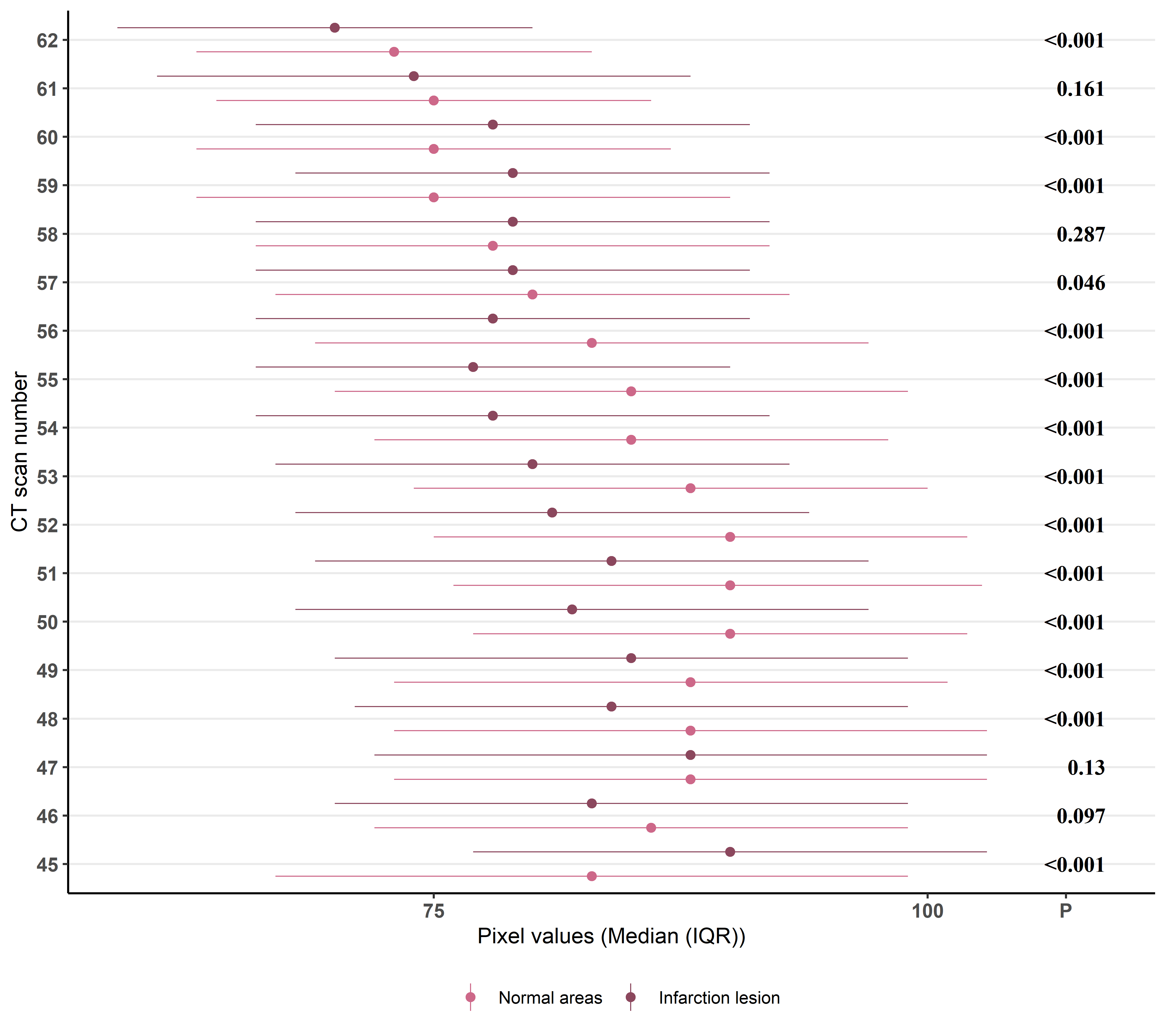


**Supplementary Figure 6:** Comparing the pixel values between the infarct and the corresponding contralateral healthy area for patient 6# under the CT scan.


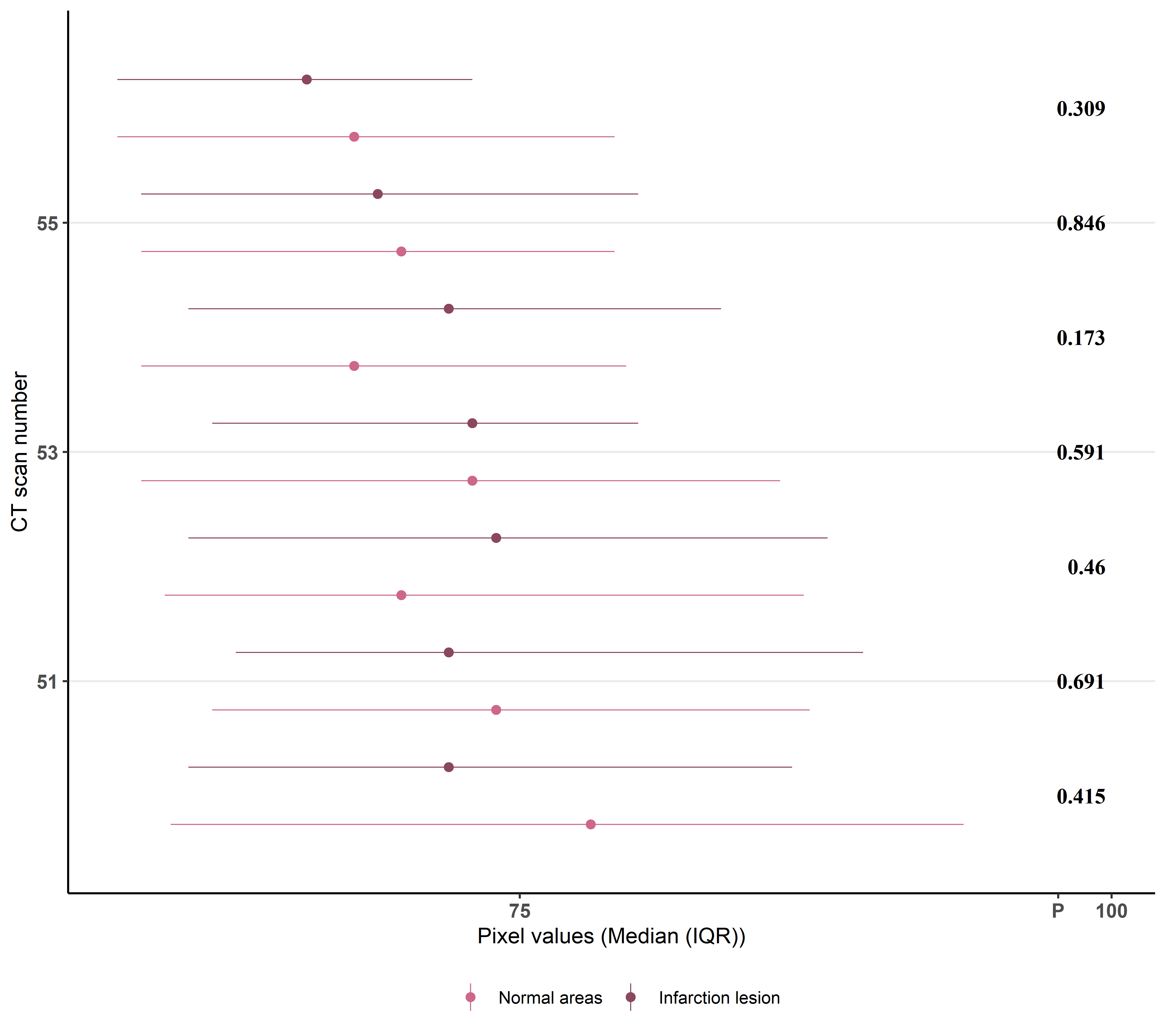


**Supplementary Figure 7:** Comparing the pixel values between the infarct and the corresponding contralateral healthy area for patient 7# under the CT scan.


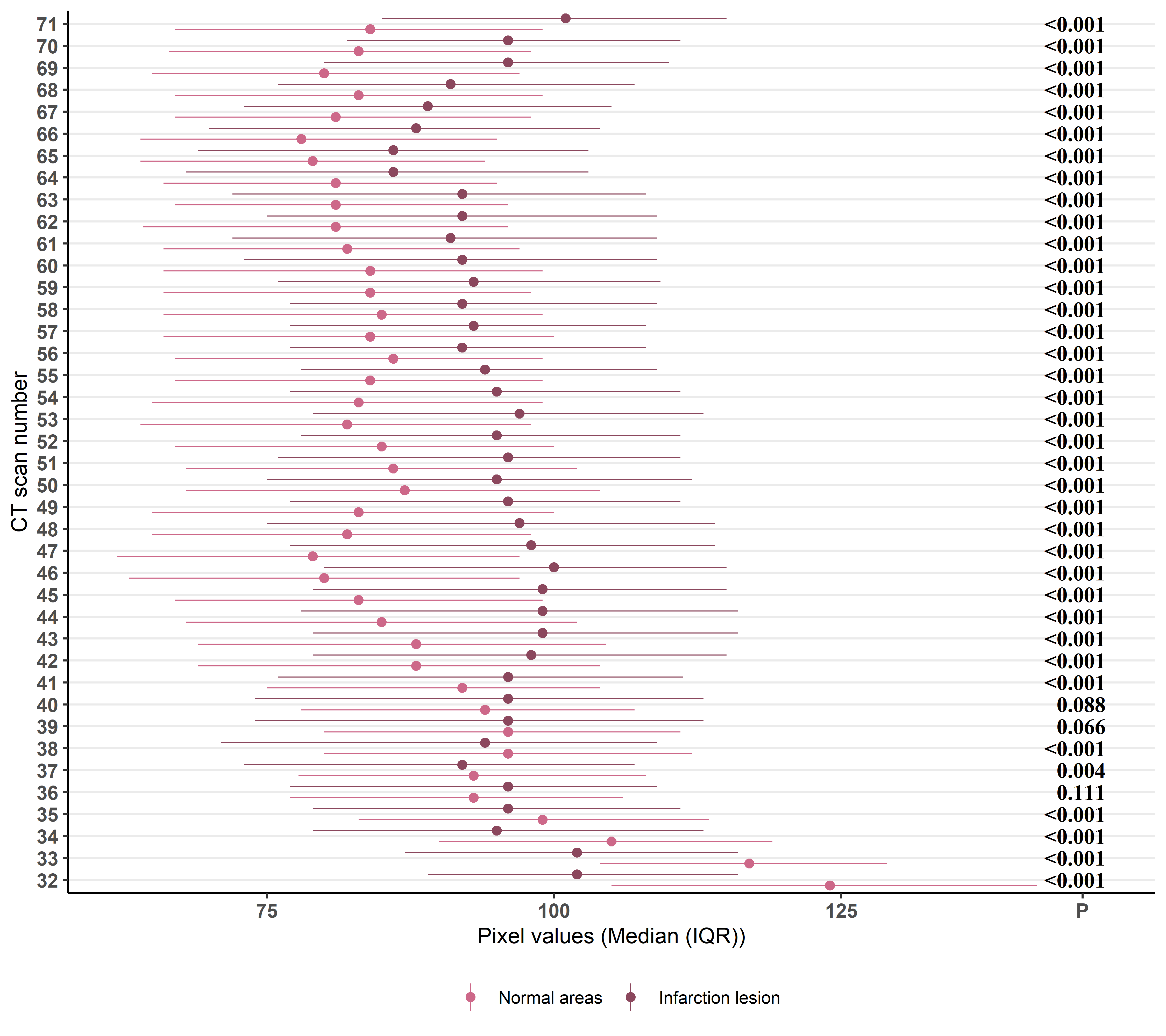


**Supplementary Figure 8:** Comparing the pixel values between the infarct and the corresponding contralateral healthy area for patient 8# under the CT scan.


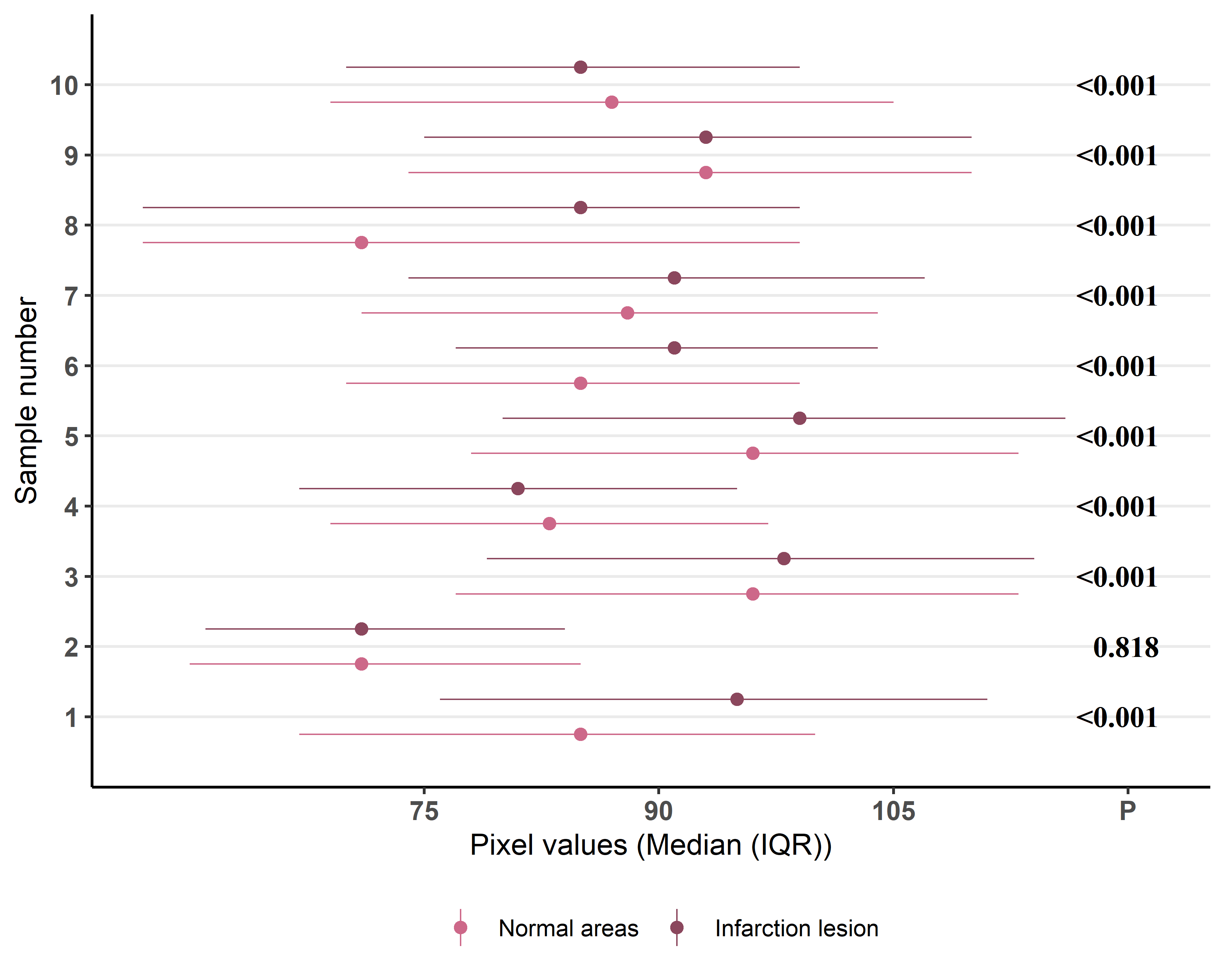


**Supplementary Figure 9:** Comparing the pixel values between the infarct and the corresponding contralateral healthy area for patient 9# under the CT scan.
